# An IR-RIME-SQP-Based Spectrum Modification Method for Shallow Surface Electromagnetic Detection Transmitting Scheme of Metal Traps in Forested Areas

**DOI:** 10.3390/s26134032

**Published:** 2026-06-25

**Authors:** Zeyu An, Xiaoyue Fan, Yuhang Wang, Tao Zhang

**Affiliations:** 1 College of Mechanical and Electrical Engineering, Northeast Forestry University, Harbin 150040, China; zeyu.an@nefu.edu.cn; 2 College of Control and Information Engineering, Northeast Forestry University, Harbin 150040, China; fanxiaoyue@nefu.edu.cn

**Keywords:** shallow surface electromagnetic detection, spectrum modification, IR-RIME-SQP, viscous remanent magnetization

## Abstract

**Highlights:**

**What are the main findings?**

- We propose an IR-RIME-SQP spectrum modification method that couples the Debye relaxation of ferromagnetic targets with soil VRM models to concentrate transmission energy into an optimal signal-to-clutter ratio (SCR) window.
- The approach achieves a targeted frequency energy retention (TFER) of 51.85%, improving upon the traditional PSO method by 16.4%, and demonstrates robustness against non-linear coil parameter drifts under hardware constraints.

**What are the implications of the main findings?**

- This targeted spectral shaping mitigates the conflict between high-frequency VRM clutter excitation and low-frequency energy waste, improving the detection capability for metal snaring traps in shallow forested areas.
- Beyond wildlife conservation, this method provides an applicable algorithmic framework for other controlled-source electromagnetic scenarios, such as unexploded ordnance clearance and underground infrastructure surveying.

**Abstract:**

In the electromagnetic detection of shallow subsurface metal snaring traps in forested areas, conventional transmission schemes such as square waves, PRBS and PSO waveforms inevitably excite severe clutter in soils exhibiting viscous remanent magnetization (VRM), which drastically degrades the signal-to-clutter ratio (SCR). To resolve this issue, this paper proposes a spectrum modification method for the transmitting scheme based on an Interval Robust hybrid RIME and Sequential Quadratic Programming (IR-RIME-SQP) algorithm. By coupling the Debye relaxation characteristics of ferromagnetic targets with the soil VRM model, the proposed method concentrates limited transmission energy into a bell-shaped frequency window near the target’s characteristic frequency. Furthermore, interval analysis is introduced to ensure robust performance against the dynamic drift of coil parameters. The feasibility of this novel transmitting scheme is validated through ablation experiments and comparative simulations. Finally, laboratory measurements demonstrate that IR-RIME-SQP provides a more rational and efficient energy allocation strategy, improving the targeted frequency energy retention (TFER) by approximately 16.4% and thereby enhancing both detection efficiency and precision.

## 1. Introduction

As the flagship species of the Asian cold-temperate forest ecosystem, the Amur tiger has been explicitly classified as an endangered species by the International Union for Conservation of Nature (IUCN). According to a 2024 report by the Northeast China Tiger and Leopard National Park (NCTLNP) Administration and the Wildlife Conservation Society (WCS) China Program, the wild Amur tiger population in this region has steadily recovered to approximately 70 individuals; however, this population size remains significantly below the theoretical environmental carrying capacity of the habitat [1]. Poaching devices, such as illegal metal snaring traps, are typically concealed beneath thin topsoil layers or leaf litter in shallow subsurface environments [2]. Given their low manufacturing cost and the fact that wildlife, including the Amur tiger, struggles to escape once ensnared [3], the accurate detection of these metal snaring traps presents a valuable research problem [4]. Compared to traditional deep mineral exploration, detecting such shallow, weak micro-targets confronts a distinct physical paradox: forest soils are rich in constituents like magnetite and exhibit intense VRM [5]. According to Néel relaxation theory, within the operational frequency band of conventional shallow subsurface detection, the induced response of such soils grows linearly with frequency, generating severe colored clutter [6]. Traditional electromagnetic induction (EMI) methods are primarily designed for deep prospecting, often assuming a uniform, low-damping background medium [7], and thus rely on low-frequency signals to achieve deep penetration. Conversely, shallow subsurface detection conventionally favors high-frequency signals to secure high resolution. However, against a VRM background, blindly elevating the transmission frequency exacerbates geological clutter, yielding no discernible improvement in the SCR [8]. Consequently, designing a transmission waveform that simultaneously suppresses the short-relaxation clutter responses of the soil while maximizing the long-relaxation Debye response signatures of ferromagnetic targets has emerged as a focus of current shallow subsurface electromagnetic detection research in forested areas.

Fundamentally, EMI detection of shallow subsurface targets is an inverse scattering problem in lossy media [9], where the design of the transmission waveform is pivotal for enhancing the system’s signal-to-noise ratio (SNR) and automatic target recognition (ATR) capabilities [10]. Early EMI systems, such as the Geonics EM-61 and Minelab series, predominantly employed bipolar square or pulse waves. While these waveforms boast simple hardware implementation, their spectral energy is highly concentrated at the fundamental frequency, with high-frequency harmonic components decaying proportionally to 1/*n* [11]. According to the theoretical frameworks established by Pasion et al. [12,13,14], the magnetic susceptibility dispersion characteristics of small ferromagnetic targets often contain abundant geometric and material information in the high-frequency band. The energy deficiency of square waves at these high frequencies consequently leads to insufficient information for accurate target feature extraction. To resolve the conflict between single-frequency energy concentration and broadband information acquisition in traditional EM detection systems, Duncan et al. [15] utilized pseudo-random binary sequences (PRBS) with fixed frequency ratios to provide multiple dominant frequencies. However, the energy dispersion inherent to PRBS results in excessively low excitation power at the target’s characteristic frequency. Li et al. [16] introduced AFEM-3, a broadband frequency-domain EM system based on an unmanned aerial vehicle platform, establishing a paradigm for subsequent EM detection systems. In recent years, energy equalization has become the mainstream trajectory in waveform design. Zhang et al. [7] proposed a spectrum modification method based on Multi-Objective Particle Swarm Optimization (MOPSO), achieving an equal energy distribution across the fundamental wave and specific high-order harmonics by reconstructing the PWM switching sequence. Following a similar mathematical logic, Yang et al. [17] developed a high-order PRBS synthesis method tailored for frequency-domain EM exploration, overcoming the energy scattering defects of traditional PRBS to achieve highly concentrated and uniform energy distribution across multiple frequencies. While these two schemes significantly enhance multi-frequency detection efficiency under white noise backgrounds or in deep underground probing, deploying an equalized energy distribution across multiple high-frequency points in VRM-dominated shallow forest environments invariably excites intense soil clutter [18], leading to a sharp decline in SCR. Furthermore, waveform designs based on deterministic models struggle to cope with the dynamic drifts of coil inductance and resistance encountered in complex field environments [19].

Mathematically, waveform design constitutes a non-convex, non-linear optimization problem subjected to strict hardware constraints. Traditional deterministic methods, such as Sequential Quadratic Programming (SQP) gradient descent, easily stagnate in local optima. Among classic meta-heuristic algorithms, Genetic Algorithms (GA) [20] exhibit weak crossover and mutation randomness; Particle Swarm Optimization (PSO) [21] suffers from slow convergence and premature stagnation when processing high-dimensional discrete switching sequences; Simulated Annealing (SA) [22] is heavily reliant on rigid parameter settings; and Differential Evolution (DE) [23] demonstrates suboptimal performance when confronting complex multi-modal problems. The RIME optimization algorithm, proposed by Su et al. [24], simulates the physical mechanisms of rime-ice formation—initial crystallization, preferential growth, and dynamic melting. It outperforms PSO and DE in escaping local optima, surpasses GA and SA in parameter setting simplicity, and excels beyond the Gray Wolf Optimizer (GWO) and Whale Optimization Algorithm (WOA) in global search capabilities for high-dimensional, multi-modal engineering problems [25]. However, the majority of existing waveform optimization schemes assume constant physical system parameters. In actual field detection, temperature drift in the coils and the load effects of magnetic soils trigger fluctuations in equivalent circuit parameters, causing the simulated waveforms to distort during physical output. Therefore, understanding interval analysis is paramount for ensuring waveform robustness under parameter variations [26], underscoring the necessity of developing a robust-optimized variant of the RIME algorithm.

Focusing on shallow subsurface metal detection in forested areas, this study presents an innovative solution to the SCR degradation problem faced by existing EMI detection techniques in magnetic soil backgrounds. Distinct from previous energy-equalization schemes primarily targeting deep minerals or large-scale unexploded ordnance (UXO), this paper proposes a spectrum modification method for the transmitting scheme based on an IR-RIME-SQP algorithm. This method integrates the Debye relaxation characteristics of ferromagnetic targets with the colored VRM clutter model of soils. Through algorithmic optimization, it dynamically concentrates limited transmission energy into a bell-shaped window possessing the optimal SCR. Additionally, to address the dynamic drifts of coil inductance and resistance inherent in field operations, interval analysis is embedded within the algorithm to execute robust optimization. Experimental results demonstrate that the excitation waveform and its spectral morphology generated post-optimization diverge significantly from preceding flat-spectrum schemes. Driven by a TMS320F28379D microcontroller under fluctuating load conditions, the proposed scheme achieves stable physical output, maximizes the extraction of the snaring trap’s characteristic signals, and robustly suppresses magnetic soil interference.

This paper first reviews the current research status of shallow subsurface EM detection and transmission spectrum optimization, exposing the limitations of conventional schemes in specific environments and the corresponding demand for improvements. Section 2 details the EM response dynamics of ferromagnetic targets and the physical mechanisms of soil VRM, establishing the theoretical foundation for subsequent waveform shaping. Building upon this, Section 2 provides a comprehensive description of the IR-RIME-SQP algorithm’s model construction, the derivation of the multi-objective fitness function, and the specific execution process of the interval robust optimization. Section 3 conducts comparative ablation based on Monte Carlo statistics and simulation experiments to evaluate the transmitting waveform’s robustness, targeted energy focusing, and fundamental wave suppression capabilities under extreme soil parameter drift conditions. Section 3 also initiates with functional laboratory measurements relying on an equivalent hardware platform, thoroughly analyzing the capabilities of IR-RIME-SQP in suppressing the fundamental frequency and broadband clutter, exciting the target’s characteristic signal, and mitigating clutter interference. This is followed by static and dynamic real-world tests on snaring traps to summarize the experimental outcomes. Finally, Section 4 and Section 5 conclude the paper, evaluate the achievements and limitations of the current study, and explore prospective directions for future research.

## 2. Materials and Methods

### 2.1. Theoretical Methods and Modeling

Based on the Néel and Debye relaxation theories, this section derives the broadband electromagnetic response models for shallow subsurface micro-ferromagnetic targets against strong VRM soil backgrounds. Subsequently, an optimal matching spectrum guided by the maximization of the SCR is established, alongside a demonstration of the physical rationale for selecting 3.5 kHz as the characteristic frequency.

#### 2.1.1. Broadband Response Models for Shallow Surface Targets and VRM Clutter

**Figure 1:**
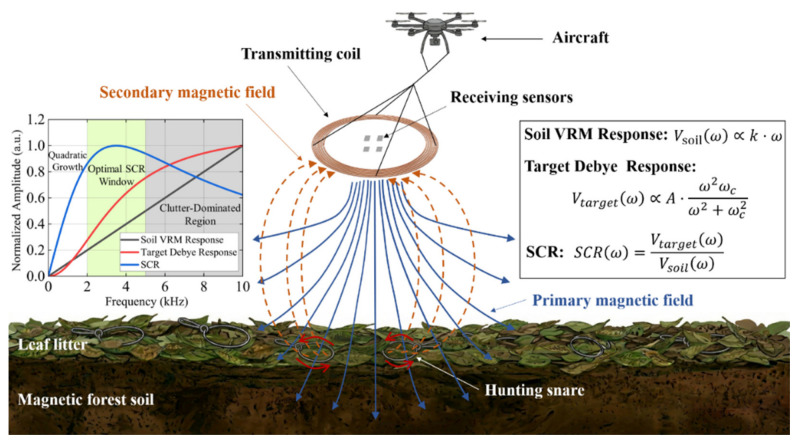
Schematic diagram of shallow subsurface EM detection in forested environments. The inset shows a plot of the SCR curve, the blue arrows represent the primary magnetic field, and the red arrows represent the secondary magnetic field.

Figure 1 illustrates the principles of shallow subsurface EM detection in forested environments. In a typical detection operation, the system primarily comprises a transmitting unit, a receiving unit, and excitation coils. Assuming the transmitting coil possesses an equivalent resistance R and an inductance L, when the output spectral voltage of the transmitting system is U(ω), the spectral amplitude ∣I(ω)∣ of the transmitting current in the coil is constrained by the complex impedance constraint of the coil.

(1) $$|I\left(\omega \right)|=\frac{|U\left(\omega \right)|}{\sqrt{R^{2}+(\omega L{)}^{2}}}$$

According to this impedance equation, as the angular frequency ω increases, the inductive reactance ωL surges, imposing severe reactive suppression on high-frequency currents. Consequently, for conventional waveforms such as square waves and PRBS, energy is predominantly concentrated at the fundamental frequency, thereby restricting detection performance. The actual total voltage signal V_actual_(ω) induced in the receiving coil comprises the effective target signal V_target_(ω) and the sum of internal thermal noise and external clutter V_noise_(ω). To evaluate detection accuracy, we define the system’s signal-to-clutter ratio SCR(ω) and the relative measurement error η(ω) as follows:

(2) $$\;SCR\left(\omega \right)=\frac{|V_{target}\left(\omega \right)|}{|V_{noise}\left(\omega \right)|}$$

(3) $$\;\eta \left(\omega \right)=|\frac{V_{actual}\left(\omega \right)-V_{target}\left(\omega \right)}{V_{target}\left(\omega \right)}|\approx \frac{1}{SCR\left(\omega \right)}$$

This formulation indicates an inverse proportionality between η(ω) and SCR(ω). Merely elevating the high-frequency excitation voltage U(ω) to overcome the ωL limitation tends to trigger severe V_noise_(ω), leading to a degraded SCR(ω) and a sharp rise in η(ω). Thus, it is imperative to investigate the differential response dynamics of micro-ferromagnetic targets versus VRM soils under alternating EM fields, which will guide the spectral modification of the transmitted waveform. According to the Néel-Arrhenius equation, the relaxation time τ_n_ of a single-domain magnetic particle exhibits an exponential relationship with its physical volume V:

(4) $${\;\tau }_{N}=\tau_{0}\exp\left(\frac{KV}{k_{B}T}\right)$$

where τ_0_ is the characteristic attempt time, K is the magnetocrystalline anisotropy constant, k_B_ is the Boltzmann constant, and T is the absolute temperature. Because the volume of magnetic particles in forest soils presents a broad, continuous distribution [27], the soil macroscopically manifests an extremely wide relaxation time spectrum. This VRM effect can be characterized by complex magnetic susceptibility. Based on the framework of Pasion et al. [12,13,14], the imaginary part of this complex susceptibility, χ″ (ω), remains almost constant across the conventional EM detection frequency range. Consequently, the amplitude of the soil clutter voltage V_soil_(ω) induced in the receiving system behaves as colored noise that grows linearly with frequency:

(5) $${\;V}_{soil}\left(\omega \right)\propto \omega \cdot \chi^{\prime \prime }\left(\omega \right)\approx k\cdot \omega$$

If a waveform spectrum attempts to inject substantial energy into the high-frequency band to obtain high resolution, this energy will excite linearly amplifying soil clutter. This growth rate significantly outpaces that of the micro-target signal, ultimately causing a drastic deterioration in the system’s SCR. Therefore, suppressing ineffective energy emission in the high-frequency regime is a physical necessity.

Conversely, ferromagnetic snaring traps possess both high electrical conductivity and high relative magnetic permeability. Their magnetic polarization tensor response M(ω) along the principal axis can be approximated by a first-order Debye relaxation model [28]:

(6) $$M(\omega )=M_{DC}+\frac{M_{\infty }-M_{DC}}{1+i\omega \tau_{c}}$$

where M_DC_ and M_∞_ denote the extreme magnetic susceptibilities at ultra-low and ultra-high frequencies, respectively, and τ_c_ is the target’s characteristic relaxation time. Given the overwhelming interference from the in-phase component of the strongly magnetic background, we isolate the quadrature component—which exhibits superior perturbation resilience—as the target’s signature signal. The amplitude of the quadrature component of the receiving voltage induced by the target’s secondary field, V_target_(ω), is derived as:

(7) $$\;V_{target}\left(\omega \right)\propto \omega \cdot Im\left\{M\left(\omega \right)\right\}=A\cdot \frac{\omega^{2}\omega_{c}}{\omega^{2}+\omega_{c}^{2}}$$

where A represents the geometric coupling coefficient of the transceiver system. This equation reveals that the target’s characteristic signal follows a quadratic growth law in the low-frequency region and asymptotically approaches a saturation constant in the high-frequency region due to the skin effect.

#### 2.1.2. Spectrum Construction Driven by SCR Maximization

Flat broadband excitation methods, such as PRBS or uniform MOPSO, aim to distribute energy evenly across multiple frequencies to achieve robust depth coverage. However, within shallow forest VRM environments, injecting equal amounts of energy into high-frequency bands triggers linearly increasing soil clutter that completely masks the target signal. Accordingly, this section proposes a spectral modification methodology for the transmission scheme predicated on SCR maximization.

By defining the frequency-domain SCR objective function SCR(ω) as the ratio of the target’s quadrature response voltage to the soil clutter voltage, we obtain:

(8) $$\;SCR\left(\omega \right)=\frac{V_{target}\left(\omega \right)}{V_{soil}\left(\omega \right)}=C_{scale}\cdot \frac{\omega \cdot \omega_{c}}{\omega^{2}+\omega_{c}^{2}}$$

where C_scale_ is a system scaling constant. Equating the first-order partial derivative of the above equation with respect to ω to zero yields:

(9) $$\frac{\partial SCR(\omega )}{\partial \omega }=C_{scale}\cdot \omega_{c}\cdot \frac{\left(\omega^{2}+\omega_{c}^{2})-\omega (2\omega \right)}{\left(\omega^{2}+\omega_{c}^{2}{)}^{2}\right.}=0\;$$

Solving this equation identifies a unique stationary point at ω = ω_c_. Mathematically, this function depicts an asymmetric bell-shaped curve. In the context of shallow forest VRM environments, energy consumed in the ω ≪ ω_c_ region fails to generate a sufficient target response, whereas energy injected into the ω ≫ ω_c_ region incites massive clutter. Therefore, the optimal transmission waveform corresponds to an asymmetric bell-shaped bandpass spectrum centered at the characteristic frequency ω_c_.

In EM induction detection, the characteristic frequency exhibits the following inverse relationship with the target’s EM parameters and geometric features [29]:

(10) $$f_{c}\propto \frac{1}{\mu_{r}\mu_{0}\sigma a^{2}}$$

For UXO featuring large target dimensions a, the a^2^ term dominates, typically yielding characteristic frequencies merely in the tens to hundreds of hertz. In contrast, the hunting snares investigated herein are typically constructed from ultra-thin braided steel wires. Their effective geometric cross-sectional radius participating in the EM eddy current circulation is exceedingly small. According to the f_c_ ∝ 1/a^2^ proportionality, this induces a blue shift in the characteristic frequency spanning several orders of magnitude. Simultaneously, the strongly ferromagnetic materials used for these snares, like high-carbon steel, feature a relative magnetic permeability μ_r_ reaching 100 to 300, which significantly compresses the skin depth. This hinders EM wave penetration into the target interior, causing the response to reach saturation prematurely—a phenomenon that conversely triggers a red shift in the characteristic frequency [30].

A study by Jiang et al. [31] concerning the optimal EM detection frequencies for micro-ferromagnetic and non-ferromagnetic targets in complex marine benthic environments indicated that the optimal detection frequency band for small tubular/flake-like metal targets resides between 1807 Hz and 5365 Hz. Consequently, the interplay between the small-size-induced blue shift and the high-permeability-induced red shift positions the characteristic frequency of snaring traps squarely within the mid-frequency band [32]. Addressing this trait, persisting with traditional flat broadband excitation methods like PRBS will inevitably result in the futile dissipation of high-frequency energy and the exacerbation of background soil clutter. This study strategically selects 3.5 kHz as the characteristic frequency, aiming to attain a globally optimal SCR by balancing the maximization of the target scattering cross-section with the suppression of high-frequency dielectric relaxation noise. Given that a full-bridge inverter under bipolar PWM modulation is bound by half-wave symmetry constraints, its output spectrum consists exclusively of odd harmonics. Hence, the fundamental frequency is optimally configured to 1.167 kHz. The precise determination of the corresponding transmitted waveform subsequently emerges as a highly significant problem in shallow subsurface forest snare detection.

### 2.2. Spectrum Modification Method Based on the IR-RIME-SQP Algorithm

The core objective of metal snare detection in forested areas lies in shifting limited transmission power from the high-frequency bands—which induce intense VRM clutter—to the target’s characteristic frequency. This section first establishes a theoretical analytical model mapping bipolar PWM switching sequences to frequency-domain current responses. Subsequently, an IR-RIME-SQP algorithm is proposed to modify the transmitted waveform’s spectrum under the physical boundary constraints of a forest environment.

#### 2.2.1. Theoretical Analysis of Spectrum Modification

In shallow EM detection systems, to avoid DC bias saturation and to maximally suppress even-harmonic interference, the PWM voltage waveform u(t) driving the H-bridge inverter must satisfy the half-wave symmetry condition. Let U_dc_ denote the DC bus voltage and f_base_ the system’s fundamental frequency. Assuming there are M voltage polarity reversal instances within a half-cycle, the corresponding discrete switching angle sequence is defined as a high-dimensional vector α = [α_1_, α_2_, …, α_M_]^T^. According to generalized Fourier series theory, the DC component and even harmonics of this waveform are strictly zero, while the voltage amplitude of its n-th odd harmonic, U_n_(α), can be determined by:

(11) $$\;U_{n}\left(\alpha \right)=\frac{4U_{dc}}{n\pi }\sqrt{{\left(\sum_{k=1}^{M}\;(-1{)}^{k+1}sin(n\alpha_{k})\right)}^{2}+{\left(\sum_{k=1}^{M}\;(-1{)}^{k}cos(na_{k})\right)}^{2}}\left(n=1,3,5,\ldots \right)$$

The above equation establishes a deterministic mapping from the asymmetric time domain switching angle sequence α to the frequency-domain amplitude and phase of specific harmonics. By inversely solving this equation, targeted spectral modification can be achieved. To suppress the linearly increasing VRM clutter at high frequencies and ameliorate energy deficiency in the low-frequency band, the ideal targeted amplitude U_n_* for each odd harmonic is designed to follow a bell-shaped bandpass distribution, perfectly consistent with the theoretically optimal SCR curve:

(12) $$\;U_{n}^{*}=U_{ref}\cdot \frac{n\cdot f_{base}\cdot f_{c}}{\left(n\cdot f_{base}{)}^{2}+f_{c}^{2}\right.}\left(n=1,3,5,7\ldots \right)$$

where U_ref_ is a normalized system gain constant. To drive the actual harmonic amplitude U_n_(α) toward the theoretical extreme U_n_*, a multi-objective fitness function F(α) is constructed to minimize the spectral residual:

(13) $$minF(\alpha )=\sum_{n\in N}\;\omega_{n}{\left(U_{n}(\alpha )-U_{n}^{*}\right)}^{2}$$

where N is the set of odd harmonic orders within the critical detection frequency band, and ω_n_ represents the weight penalty coefficient at different frequency points. Throughout the algorithmic process, this coefficient matrix forcefully guides energy concentration toward the target’s characteristic frequency.

When solving this non-convex objective function, the switching angle sequence α is restricted by hardware limitations and cannot assume arbitrary values across the entire space. To preserve the temporal monotonicity of the half-wave, all independent switching angles within the half-cycle interval must strictly increase. Furthermore, to prevent shoot-through short circuits on the same bridge arm, a dead time Δt_dead_ of 1 microsecond is inserted between adjacent switching state flips. Projecting this into the phase angle domain yields the following minimum phase span inequality constraints:

(14) $$\;\alpha_{k}-\alpha_{k-1}\geq \Delta \alpha_{min}=2\pi f_{base}\cdot \Delta t_{dead}\left(k=2,3,\ldots ,M\right)$$

(15) $$\alpha_{1}\geq \frac{\Delta \alpha_{min}}{2},\pi -\alpha_{M}\geq \frac{\Delta \alpha_{min}}{2}$$

The introduction of Δt_dead_ in these constraints escalates the difficulty of constructing a viable solution space. Traditional deterministic algorithms are prone to stagnating in local optima. Concurrently, field environments induce non-linear fluctuations in the L and R. Consequently, this study employs the IR-RIME-SQP algorithm to execute the spectrum modification.

#### 2.2.2. Spectrum Modification Methodology Using the IR-RIME-SQP Algorithm

The IR-RIME-SQP algorithm is utilized here to achieve the spectral modification of the transmitted current. The switching angle sequence within a half-cycle is defined as the algorithm’s optimization variable X = [x_1_, x_2_, …, x_N_]^T^, where N represents the optimization dimension. To prevent bridge-arm shoot-through short circuits when the microcontroller drives the H-bridge inverter, the particle positions must satisfy the monotonic increasing property and the dead time limits, which are translated into the following linear inequality constraints:

(16) $$\left[\begin{array}{ccccc} 1 & -1 & 0 & \ldots & 0\\ 0 & 1 & -1 & \ldots & 0\\ \vdots & \vdots & \ddots & \ddots & \vdots \\ 0 & 0 & 0 & 1 & -1 \end{array}\right]\left[\begin{array}{c} x_{1}\\ x_{2}\\ \vdots \\ x_{N} \end{array}\right]\leq \left[\begin{array}{c} -\Delta t_{min\;\_norm}\\ -\Delta t_{min\;\_norm}\\ \vdots \\ -\Delta t_{min\;\_norm} \end{array}\right]$$

where Δt_min_norm_ is the normalized minimum physical dead time span. Environmental perturbations are translated into continuous bounded intervals: L ∈ [L_min_, L_max_] and R ∈ [R_min_, R_max_]. To avoid the dimensionality curse associated with exhaustive continuous space evaluation, four physical boundary corner cases are extracted to form a discrete boundary set: Θ = {(L_nom_, R_nom_), (L_min_, R_min_), (L_max_, R_max_), (L_min_, R_max_)}. For any single operating condition k ∈ Θ, a multi-objective cost function Cost_k_(X) is constructed:

(17) $$Cost_{k}(X)=f_{1}^{\left(k\right)}(X)+\lambda \cdot f_{2}^{\left(k\right)}(X)+f_{3}^{\left(k\right)}(X)+P_{shape}$$

where f_1_^(k)^(X) is the targeted energy maximization objective, f_1_^(k)^(X) = −J_3_^(k)^(X), which forces the emitted energy to gather at the characteristic frequency; f_2_^(k)^(X) is the SCR-matching objective, f_2_^(k)^(X) = −J_3_^(k)^(X)/(∑_i≠3_ J_i_^(k)^(X) + ϵ, designed to suppress ineffective emitted energy outside the target frequency band; and f_3_^(k)^(X) is a safety limit penalty function, where f_3_^(k)^(X) = Φ(I_peak_^(k)^ − I_limit_), which triggers an exponential penalty if the simulated peak current exceeds the H-bridge safety margin; and P_shape_ is a bandpass topology penalty that restricts the fundamental wave energy from surpassing that of the third harmonic. Therefore, this study constructs a final interval robust evaluation function Z_robust_(X) based on a minimax and variance regularization hybrid evolution approach [33]:

(18) $$minZ_{robust}(X)=\underset{k\in \Theta }{max}\;(Cost_{k}(X))+\gamma \sqrt{\frac{1}{4}\sum_{k\in \Theta }\;{\left(Cost_{k}(X)-\overset{\_\_\_\_\_\_}{Cost}\right)}^{2}}$$

**Figure 2:**
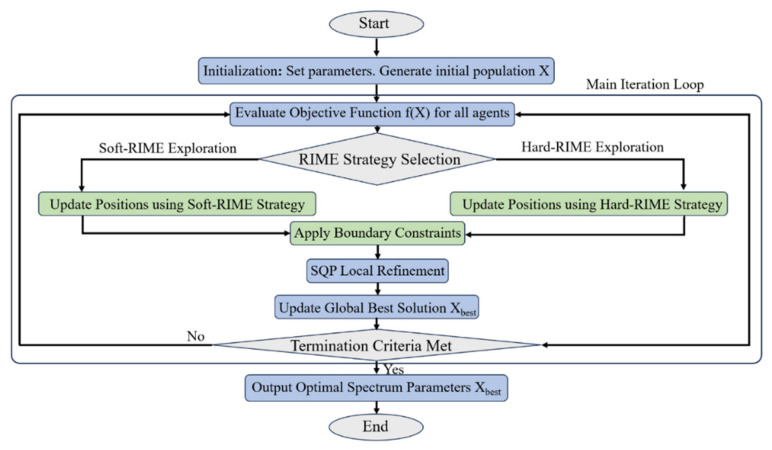
IR-RIME-SQP Solution Flowchart.

The first half of this function compels the algorithm to focus heavily on the most severe detuning conditions to elevate baseline performance, while the penalty term γ in the latter half acts to eliminate fragile solutions that exhibit erratic jumps across different soil physical boundaries. Because the solution space mapped by the objective function Z_robust_(X) is multi-modal and non-convex, a two-stage non-linear solving paradigm is adopted. Figure 2 illustrates the IR-RIME-SQP solution flowchart.

The first stage involves global robust basin exploration based on IR-RIME. RIME possesses exceptional high-dimensional topological penetration capabilities [34], with 15 normalized switching angles serving as the search dimension variables within the half-cycle. The algorithm regulates the evolutionary process via an attachment coefficient E that decays dynamically with the iteration number it. In the early iterations (r_1_ < E), the hard-rime puncture strategy is activated, permitting particles to execute large-step leaps to escape local minimum traps:

(19) $$X_{i,j}^{new}=X_{best,j}+R\cdot (ub_{j}-lb_{j})\cdot rand()$$

(20) $$E=\sqrt{it/Max_{-}Iter}$$

where the rime factor R decays alternately with environmental temperature differences, guiding the solutions to perform bidirectional positive and negative punctures around the historical robust optimal guide X_best_. When r_1_ ≥ E, the system transitions to soft-rime micro-exploration. The probability of a particle being selected for resetting is proportional to its current normalized robust fitness value, and it is assigned a differentiated centripetal convergence step:

(21) $$X_{i,j}^{new}=X_{best,j}+(rand()-0.5)\cdot 2\cdot \frac{ub_{j}-lb_{j}}{it+1}$$

This equation compels adverse marginal solutions to rapidly converge toward the currently discovered robust optimal solution. After each position update, the algorithm enforces sequential sorting to strip away logically invalid solutions.

The second stage based on local fine-grained locking and linear temporal inequality constraints based on SQP. Given the stochastic stagnation that probabilistic meta-heuristic algorithms like RIME may encounter when approaching the ultimate optimal solution, the global historical optimal array X_best_ output from the first stage serves as the initial base point for the SQP solver. By establishing a positive-definite approximation of the objective function’s Lagrangian–Hessian matrix at each step, SQP executes a deterministic convergence cut along the steepest descent trajectory [35]. During the microcontroller’s driving of the H-bridge inverter, a dead time redline of Δt_min_ = 8.0 μs is established to prevent bridge-arm shoot-through caused by insudead timedead-time. When translated into the normalized parameter min_dt_norm, a high-dimensional linear inequality constraint equation A_ineq_⋅X ≤ b_ineq_ must be strictly imposed within the SQP’s external model:

(22) $$\left[\begin{array}{ccccc} 1 & -1 & 0 & \cdots & 0\\ 0 & 1 & -1 & \cdots & 0\\ \vdots & \vdots & \ddots & \ddots & \vdots \\ 0 & 0 & \cdots & 1 & -1 \end{array}\right]\left[\begin{array}{c} X_{1}\\ X_{2}\\ \vdots \\ X_{9} \end{array}\right]\leq \left[\begin{array}{c} -min_{d}t_{n}orm\\ -min_{d}t_{n}orm\\ \vdots \\ -min_{d}t_{n}orm \end{array}\right]$$

The SQP solver utilizes the constrained Jacobian matrix to repel all adjacent switching points beyond the dead time redline. The resulting globally robust floating-point optimal solution is reconstructed and mapped into the 50 MHz clock domain, undergoing half-wave symmetric expansion to generate a lookup table sequence comprising 29 level-toggle instruction points. Driven by this polled compare-register array, the IR-RIME-SQP achieves its designated function: transitioning from SCR-guided non-linear spatial optimization to practical spectrum modification of the transmitted waveform, while maintaining robust resilience against the dynamic conditions of the forested environment.

## 3. Results

### 3.1. Comparative Simulations and Analysis

#### 3.1.1. Comparative Ablation Study

To verify the effectiveness and robustness of the proposed IR-RIME-SQP approach in complex shallow forest surface detection environments, this section conducts comparative ablation experiments considering both the VRM soil background and extreme environmental temperature drifts. Because temperature variations and magnetic soils in forest environments induce non-linear fluctuations in the transmitting coil parameters, conventional deterministic optimization methods are prone to performance degradation. Consequently, this section evaluates the performance of the proposed algorithm alongside its variants through Monte Carlo statistical simulations.

The system’s DC bus voltage is configured to V_bus_ = 20.0 V, with a fundamental frequency of f_base_ = 1.167 kHz. The transmitting coil’s nominal parameters are set to L_nom_ = 151 μH and R_nom_ = 0.25 Ω, with a minimum dead time of 8.0 μs. For stress-testing purposes, the parameter drift interval for L is set to [65%, 135%] of its nominal value, while the drift interval for R is set to [45%, 150%]. Under these operating conditions, 60 independent Monte Carlo simulations were executed for the Classic RIME, IR-RIME, RIME-SQP, and IR-RIME-SQP algorithms. The population size was configured to 30, and the RIME evolution was run for 50 generations before transitioning to the SQP solver. To objectively assess worst-case performance under parameter fluctuations, the evaluation benchmark is defined as the true robust cost function, which signifies the maximum fitness penalty extracted under the four-corner boundary perturbation conditions.

**Figure 3:**
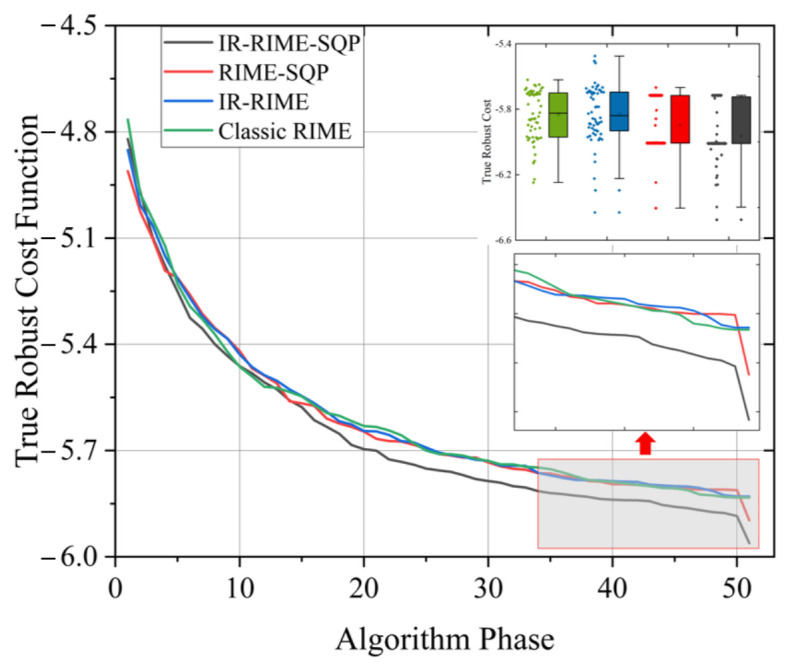
Results of the ablation experiment. The inset shows the boxplots and a close-up view.

Figure 3 depicts the convergence trajectories and boxplots derived from the 60 independent runs. During the meta-heuristic exploration phase of generations 1–50, the convergence trajectories of Classic RIME, IR-RIME, and RIME-SQP entangle and linger at relatively high cost levels. This indicates their difficulty in steadily converging toward the true minimax surface when navigating vast parameter drift spaces. IR-RIME-SQP, leveraging its interval variance regularization mechanism, maintains a convergence trajectory consistently lower than the others throughout the exploration phase, actively driving the population toward robust valleys that are insensitive to parameter drift. Upon introducing the SQP operator, which possesses formidable local exploitation capabilities at generation 51, the cost of IR-RIME-SQP plunges rapidly, firmly locking onto the optimal switching sequence. This demonstrates that under severe stress testing, neither heuristic random searches nor single-point-reliant gradient descent methods can establish a sustained convergence advantage within a highly non-linear robust cost space.

In the boxplots, the scatter points for the two SQP-assisted algorithms exhibit distinct strip-like aggregations at the upper and lower edges of the boxes, and their interquartile range boxes share highly similar vertical distributions. This occurs because the SQP solver, in its drive to amplify the energy of the third harmonic, continually compresses the intervals between adjacent switching angles until the hard dead time constraints are triggered. Consequently, the solution space is strictly confined, prompting the solutions to accumulate at local minimum boundaries dictated by hardware limitations. RIME-SQP, which is trained exclusively on nominal parameters while disregarding extreme boundary conditions, occasionally achieves lower cost limits in certain random runs; thus, the lower whisker of its boxplot extends further down than that of IR-RIME-SQP. However, this lower limit is an artifact of overfitting, rendering the derived solution highly susceptible to failure under actual physical parameter fluctuations. IR-RIME-SQP not only sustains a primary performance level comparable to that of RIME-SQP but also features several lower-cost outliers below its interquartile box. This highlights that under the IR mechanism, the algorithm successfully leverages high-quality robust initial solutions formed in earlier stages, coupling them with SQP’s local cutting capability to secure robust optimal timing sequences that excel even under severe operating conditions. This further validates the feasibility of IR-RIME-SQP in overcoming the dead time traps to which traditional methods succumb when encountering soil VRM.

To further demonstrate the repeatability and stability of the IR-RIME-SQP algorithm, a more detailed statistical analysis was conducted focusing on the energy distribution at key frequency points. Under the same hardware constraints and parameter drift conditions, 50 independent runs of the IR-RIME-SQP algorithm were executed. The statistical results of the current amplitudes at the target characteristic frequency and the fundamental frequency were extracted. The analysis reveals that across the 50 runs, the algorithm consistently concentrates high-frequency energy at 3.5 kHz, maintaining a high median current amplitude with a tight data distribution and small standard deviation. Conversely, the fundamental current amplitude is stably suppressed to a low range, effectively minimizing energy waste. This statistical evidence further corroborates the algorithmic stability and repeatability of IR-RIME-SQP in robust spectral energy allocation when facing complex non-linear constraints.

#### 3.1.2. Comparative Simulation Analysis

To further validate the effectiveness and robustness of the IR-RIME-SQP spectrum modification method in shallow forest environments, comparative simulation experiments were conducted in MATLAB R2024a. In the simulations, the parameter drift interval for L was defined as ±5%, and that for R as −5% to +20%. The waveform comprised 15 switching angles driven by a 20 V DC bus voltage. The evaluated transmission schemes included a traditional 50% duty cycle square wave, PRBS, PSO, and IR-RIME-SQP.

**Figure 4:**
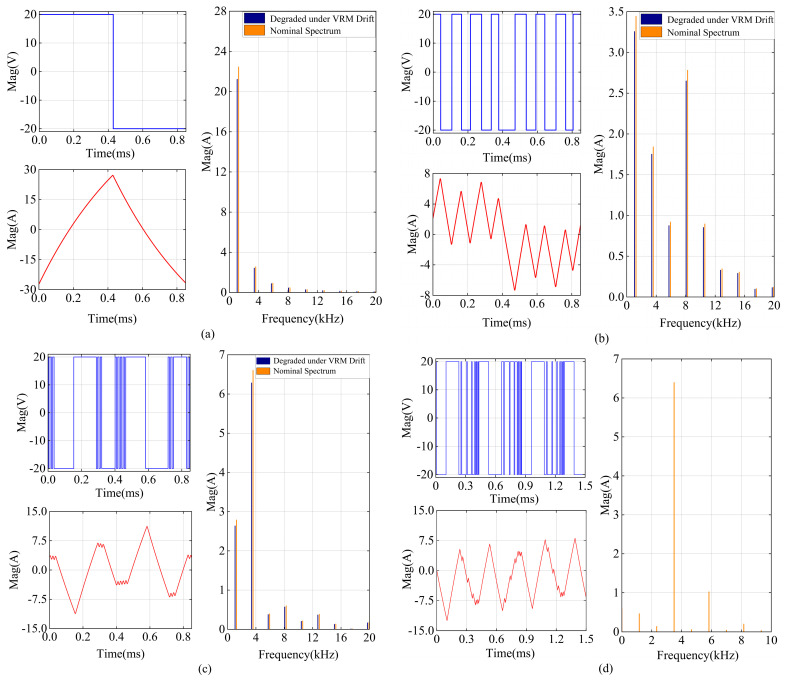
Simulation results for different transmission schemes. (**a**) Traditional square wave. (**b**) PRBS. (**c**) PSO. (**d**) IR-RIME-SQP.

The simulation results for the traditional square wave with a 50% duty cycle are shown in Figure 4a. Lacking switching angles for internal modulation, the coil current exhibits massive triangular wave oscillations with peak currents exceeding 20 A, which severely surpasses the load capacity of conventional detection power supplies. Moreover, its spectral energy is intensely concentrated at the fundamental frequency, causing the current amplitude at the critical third harmonic to be severely attenuated. This failure to sustain effective excitation energy in the target frequency band severely restricts detection efficiency.

The objective of the PRBS is to scatter energy across a broad frequency band to achieve multi-frequency coverage. However, as illustrated in Figure 4b, the current waveform and spectrum reveal an overly dispersed energy distribution. The current response at 3.5 kHz is heavily suppressed, with vast amounts of energy consumed by the fundamental wave and non-target high-order harmonics. In a strong VRM magnetic soil background, injecting substantial energy into high-frequency bands universally excites intense geological clutter that grows linearly with frequency. This deteriorates the SCR and entirely masks the faint signals emitted by small-sized metal targets.

PSO can steer limited transmission energy toward the target’s characteristic frequency. As observed in the spectrum of the PSO-optimized waveform in Figure 4c, this algorithm essentially manages to concentrate energy at the third harmonic, achieving a targeted current amplitude of 6.60 A under nominal conditions. Nevertheless, PSO exhibits insufficient suppression of the fundamental wave energy when tackling high-dimensional, multi-objective non-convex optimization problems. Constrained by the timing of 15 switching angles, PSO frequently stagnates in local optima. While it manages to amplify the third harmonic, it fails to suppress the fundamental wave, yielding a fundamental amplitude of 2.79 A under nominal conditions and 2.64 A under drift conditions. In the physical context of shallow forest detection, this low-frequency fundamental wave is incapable of fully exciting the target’s polarization response, leading to a waste of limited DC bus power and diminished detection efficiency.

Figure 4d presents the results of IR-RIME-SQP. In the time domain, the current waveform satisfies the requirement for third-harmonic dominance, and no excessively narrow pulses occur between switching actions, fully complying with MCU dead time constraints. In the frequency domain, the fundamental current amplitude is 0.46 A, while the third harmonic amplitude is 6.39 A. Evidently, IR-RIME-SQP shifts spectral energy away from bands prone to triggering intense VRM noise and concentrates it precisely near the characteristic frequency. Because IR-RIME-SQP achieves robust cost convergence across parameter drift boundaries, the third-harmonic amplitude at the characteristic frequency remains stable despite environmental fluctuations.

Therefore, based on the comparative simulations and analyses above, the IR-RIME-SQP scheme demonstrates superior robustness in main frequency distribution and spectral energy allocation under parameter-drift environments compared to traditional transmission schemes. This fundamentally proves the practical feasibility of this novel spectrum modification method for electromagnetic transmission schemes.

To fully justify the algorithmic choice, comparative simulations employing other established meta-heuristic methods, namely the GA and DE, were also conducted under the identical hardware constraints. In the time domain, although the voltage waveform generated by GA satisfied the dead time limits, its switching actions exhibited highly fragmented and dense characteristics, preventing the integral current from forming a smooth triangular wave. In the frequency domain, GA became trapped in local optima when navigating the high-dimensional, non-convex space with hard constraints; it yielded an effective current amplitude of 5.54 A at the 3.5 kHz characteristic frequency but retained a high amplitude of 3.16 A at the 1.167 kHz fundamental frequency. Similarly, the DE-optimized waveform displayed severe irregular pulsations at the peaks and troughs of the time domain current. Spectrally, DE achieved 6.06 A at 3.5 kHz and 3.24 A at 1.167 kHz. Evidently, traditional algorithms like GA, DE, and PSO frequently stagnate in dead-zone penalty traps, failing to simultaneously fulfil the dual objectives of aggressively suppressing the fundamental wave while maximizing the third harmonic.

### 3.2. Laboratory Experiments

**Figure 5:**
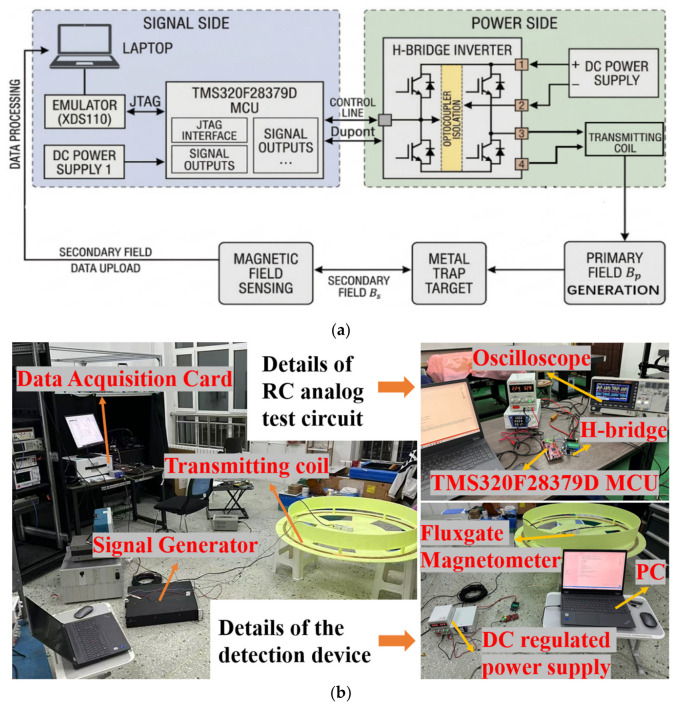
Transmitter and receiver modules. (**a**) Schematic diagram. (**b**) Actual measurement setup and RC analog test circuit.

To further validate the effectiveness and spectrum modification capability of the proposed scheme for detecting shallow subsurface metal snaring traps in forested areas, this section establishes a hardware test platform based on the TMS320F28379D microcontroller and an H-bridge inverter. As depicted in Figure 5, the transmitting and receiving modules primarily comprise the signal side, power side, transmitting coil, and receiving apparatus.

In a frequency-domain electromagnetic (FDEM) detection system, the PWM voltage V_in_(t) output by the H-bridge inverter is applied to a transmitting coil with an inductance L and internal resistance R. Because the coil’s inductive reactance far exceeds its internal resistance within the target frequency band, the macroscopic current I_L_(t) acts as an approximate time integral of the input voltage. To prevent high currents and thermal issues triggered by the low-impedance characteristics of physical transmitting coils under high-power drives, this experiment introduces a high-impedance RC analog test circuit based on the principle of circuit duality. By strictly matching the RC circuit’s time constant τ_RC_ = R_test_ C_test_ with the true RL coil’s time constant τ_RL_ = L/R_coil_, the measured capacitor voltage waveform becomes highly equivalent to the actual coil current in both morphology and phase. Based on prior calibration parameters, a 1000 Ω aluminum-housed resistor and a 680 nF metallized polypropylene film capacitor were selected, yielding an actual time constant of 680 μs. This configuration satisfies the system’s integration requirements while capping the maximum steady-state short-circuit current at under 20 mA. Data acquisition utilized a pseudo-differential measurement method with dual oscilloscope probes to extract the equivalent analog current waveform for FFT analysis.

**Figure 6:**
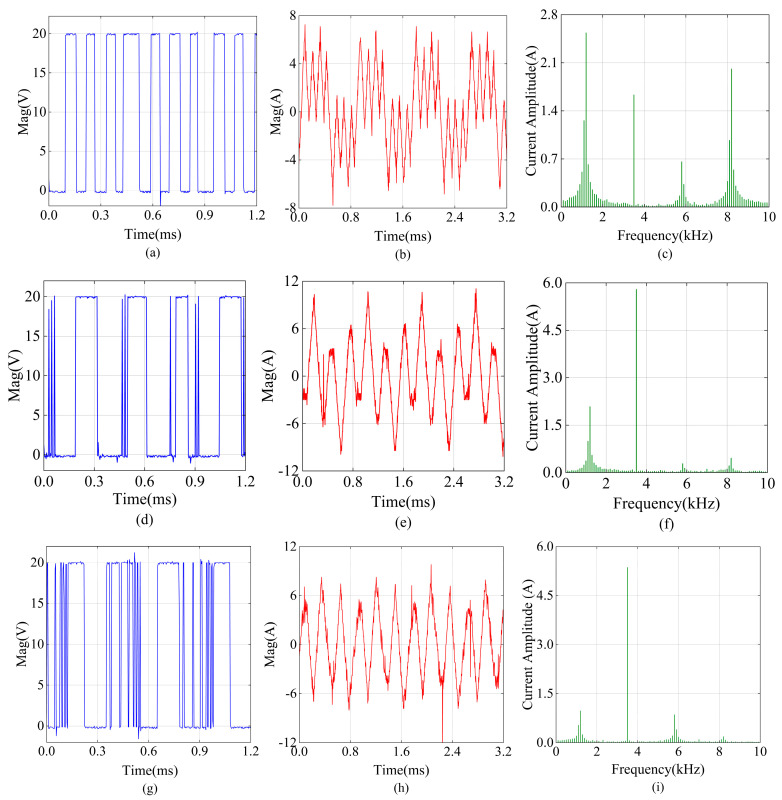
Experimental results for different transmission schemes. (**a**) PRBS voltage waveform. (**b**) PRBS current waveform. (**c**) PRBS current spectrum. (**d**) PSO voltage waveform. (**e**) PSO current waveform. (**f**) PSO current spectrum. (**g**) IR-RIME-SQP voltage waveform. (**h**) IR-RIME-SQP current waveform. (**i**) IR-RIME-SQP current spectrum.

Initially, the PRBS experiment was conducted. Figure 6a displays the time domain voltage waveform. The single-arm bridge voltage driven by PRBS exhibits steep-edged and relatively regular square-wave transitions, with a peak-to-peak voltage of approximately 21.56 V and no discernible overshoot. Figure 6b illustrates the time domain current waveform. Because PRBS has fewer polarity reversal points within a half-cycle and varying pulse widths, the positive and negative voltage pulses are prone to rapid, mutual charge neutralization and cancelation within the RC low-pass integrating circuit. This physical inertia prevents the circuit from achieving highly efficient and sustained accumulation of magnetic field energy. As shown in the current spectrum in Figure 6c, PRBS retains a high amplitude of approximately 2.53 A at the fundamental frequency of 1.166 kHz, but this amplitude attenuates to roughly 1.63 A at the crucial 3.5 kHz characteristic frequency. Moreover, its energy is widely dispersed across the entire frequency band, with approximately 2.0 A of current energy still present at high frequencies such as 8.166 kHz. Although the broadband energy equalization strategy of PRBS provides broad coverage, this indiscriminately leaked full-band energy excites intense VRM clutter in strongly magnetic soil backgrounds, burying the faint signals of small targets and hindering SCR improvements.

Meta-heuristic algorithms such as PSO can effectively overcome the energy dispersion problem of PRBS, achieving specific spectral modifications through iterative optimization. The resulting current spectrum is shown in Figure 6f. PSO successfully elevates the current amplitude at 3.5 kHz to approximately 5.79 A. The switching angle sequence generated by the algorithm’s optimization is: [200, 600, 1000, 1400, 1800, 7721, 14476, 14876, 15276, 15676, 16076, 20029, 20429, 20829, 21229, 21628, 22028, 22428, 22828, 23228, 29150, 35904, 36304, 36704, 37104, 37504, 41457, 41857, 42257, 42657]. These 30 switching angles contain numerous high-frequency, continuous flips with extremely short intervals. Due to the strict limits of dead time, the single-arm bridge voltage time domain waveform in Figure 6d reveals instances where the falling edge encroaches upon the rising edge, failing to reach the calibrated 20 V level. In high-dimensional, non-convex optimization, traditional PSO frequently stagnates in local optima. To minimize calculation errors in the frequency-domain objective function, the algorithm outputs exceedingly narrow pulses approaching the hardware’s physical response limits. These ultra-narrow pulses cannot establish effective currents in an inductive load; instead, they repeatedly excite the circuit’s parasitic inductance and capacitance, inducing resonant overshoots during voltage steps. Consequently, the maximum voltage reaches 22.05 V, and the peak-to-peak voltage hits 22.90 V. The equivalent current waveform in Figure 6e fails to form satisfactory linear integration, exhibiting high-frequency oscillations with a peak-to-peak value of 23.1 A. High di/dt transients in the inverter generate significant back electromotive force (EMF), exacerbating thermal losses in the bridge switches. Its current spectrum indicates that, although PSO significantly enhances the 3.5 kHz amplitude, a high residual amplitude of 2.08 A remains at the fundamental frequency, alongside a high-frequency energy leak of approximately 0.46 A at the seventh harmonic. Thus, when tackling multi-objective optimization with rigid constraints, PSO struggles to balance rational global energy allocation with time domain waveform smoothness.

Testing the IR-RIME-SQP algorithm reveals a more balanced performance in waveform structure and energy allocation. The optimal switching angle sequence generated by the proposed algorithm is: [250, 5157, 11610, 12654, 13072, 15461, 15861, 17880, 18280, 19427, 19827, 20228, 20628, 21028, 21428, 21679, 26586, 33039, 34083, 34501, 36890, 37290, 39309, 39709, 40856, 41256, 41657, 42057, 42457]. Correlating this with the single-arm bridge voltage time-domain waveform in Figure 6g, these 29 reversal points exhibit a non-linear clustered distribution, effectively preventing the falling edge encroachment caused by ultra short, continuous high-frequency flips. The measured voltage waveform switches smoothly with a peak-to-peak value of 22.71 V. The current time domain waveform in Figure 6h demonstrates that the integrated current forms a relatively continuous and smooth pseudo-triangular wave with a peak-to-peak value of 16.2 A. This proves that the sequence achieves high electrical-to-magnetic energy conversion efficiency while sustaining a unidirectional magnetic field build-up, thereby reducing dynamic switching losses in the inverter. In bipolar PWM modulation, the fundamental frequency inherently occupies the vast majority of the square wave’s expansion energy. The current spectrum in Figure 6i indicates that while the IR-RIME-SQP amplitude at 3.5 kHz quantitatively matches the high level of PSO, the proposed algorithm leverages superior global optimization capabilities to aggressively suppress the fundamental energy down to 0.97 A. The system effectively shifts limited energy from the low-frequency band exclusively to the target detection band. Furthermore, IR-RIME-SQP presents a discrete amplitude component of approximately 0.85 A at the 5th harmonic. This represents a natural energy spillover to higher-order odd harmonics that occurs when compressing fundamental energy, shifting it to the third harmonic, and solving the problem globally. As seen in Figure 6i, aside from the characteristic frequency peak and the fifth harmonic, the entire broadband background approaches a near-zero noise floor. A single, discrete high-frequency harmonic spike is insufficient to excite continuous geological clutter, and its low-noise-floor characteristic successfully suppresses VRM clutter excitation.

Combining the measured and simulated spectra, it is notable that the overall measured equivalent current amplitudes across all schemes are slightly lower than their theoretical simulated values. This inherent amplitude attenuation primarily stems from the loss of effective dead time duty cycle in the hardware programming, the dynamic conduction voltage drop of the switches, and parasitic damping. Nonetheless, the overall energy distribution laws align with simulation expectations. Traditional schemes have distinct advantages in broadband coverage or specific frequency enhancement, but they suffer from energy dispersion when confronting VRM soil environments and hardware clock constraints. Through multi-objective optimization, IR-RIME-SQP resolves these issues while ensuring time-domain waveform smoothness and safeguarding hardware security.

**Figure 7:**
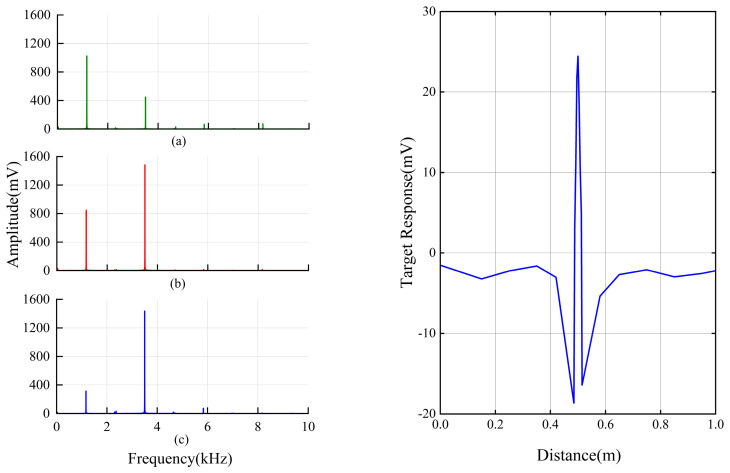
FFT results for the three transmission schemes and the displacement response curve of the animal trap. (**a**) PRBS. (**b**) PSO. (**c**) IR-RIME-SQP.

To evaluate the practical physical performance of the different transmission schemes, the MCU-output switching sequences were loaded into the H-bridge inverter to directly drive the physical transmitting coil. The primary field signals established by the transmitted waveforms were acquired using a fluxgate magnetometer placed at the center of the coil. According to Figure 7a, the response amplitudes of the PRBS scheme at 1.166 kHz and 3.5 kHz are 1023.17 mV and 447.19 mV, respectively. A massive amount of energy is wasted at the fundamental frequency, resulting in dismal target excitation efficiency. According to Figure 7b, the response amplitudes of the PSO scheme at 1.166 kHz and 3.5 kHz are 845.51 mV and 1482.54 mV, respectively. While it injects significant energy into the characteristic frequency, its confinement within local optimum traps leads to insufficient fundamental wave suppression; the prominent low-frequency fundamental wave consumes excessive power without effectively exciting the target response. According to Figure 7c, the response amplitudes of the IR-RIME-SQP scheme at 1.166 kHz and 3.5 kHz are 313.91 mV and 1435.66 mV, respectively. It preserves a highly concentrated target amplitude while drastically repressing the fundamental wave. Overall, under the premise of satisfying physical hardware constraints, the spectral characteristics of the proposed transmission scheme are substantially ameliorated.

To further elucidate this capability, a reception test was conducted utilizing the excitation signal from Figure 7c. The metal snaring trap was moved along the coil’s diameter from one edge to the other to obtain continuous spatial response characteristics. Response data were collected at 20 discrete spatial positions, with the effective received signal defined as the difference between the received signal and the background signal at the 3.5 kHz characteristic frequency. Because hunting snares are typically constructed from ultra-thin wound high-carbon steel wires, their effective cross-sectional area participating in the electromagnetic eddy current circulation is exceedingly small, causing the induced secondary field to suffer severe vertical attenuation. Therefore, this experiment adopted extreme near-field conditions for spatial profile scanning to verify whether the system could accurately capture and reconstruct the target’s response trajectory when stimulated by the proposed transmission scheme. The graph on the right side of Figure 7 illustrates the displacement response curve of the snaring trap. When positioned far from the fluxgate, the response remains between −5 mV and 0 mV. As the target gradually approaches the center, the response first drops to approximately −18.7 mV due to the physical obstruction of the sensor, followed by a sharp non-linear escalation, reaching a peak of 24.4 mV at the coil’s exact center. As the target moves toward the opposite edge, the response intensity decays symmetrically. This spatial distribution characteristic conforms to the theoretical distribution of induced electromagnetic fields. Ultimately, the spectral energy distribution of the proposed scheme is vastly more rational, enabling the precise excitation and identification of the target’s characteristic response. Thus, the real-world metal snaring trap detection experiment provides further verification of the efficacy of the IR-RIME-SQP spectrum modification scheme.

To experimentally validate the robustness of selecting 3.5 kHz as the characteristic frequency, a sensitivity analysis was conducted based on the physical properties of the target and the received response data. In actual field conditions, metal snaring traps are typically constructed by twisting multiple fine steel wires to ensure flexibility and tensile strength. To accurately replicate this, a bundle of high-carbon steel wires with diameters ranging from 0.6 mm to 1.7 mm was used as the analog target in our experiments. High-carbon steel possesses both high electrical conductivity and high relative magnetic permeability. As discussed in Section 2.1.2, the interaction between its extremely small effective eddy current cross-sectional area and the skin effect induced by high magnetic permeability determines that its Debye relaxation peak inherently falls within the mid-frequency band.

To precisely confirm that the Debye relaxation peak of this specific snare aligns with 3.5 kHz, FFT peak extraction and sensitivity analysis were performed across different target frequency windows based on the reception test data. When the target characteristic frequency was set to exactly 3.5 kHz with a 20 Hz search tolerance, the extracted spatial response curve was highly symmetrical, reaching a maximum amplitude of 24.4 mV at the center, as shown in Figure 7c, right panel.

However, when the extraction frequency was slightly shifted to 3.47 kHz or 3.53 kHz with the same tolerance, the spatial distribution curves immediately underwent severe distortion, and the peak responses plummeted to 0.65 mV and 0.53 mV, respectively.

Furthermore, examining other principal frequencies in the transmission spectrum—such as the fundamental frequency and the fifth harmonic—yielded significantly weaker peak responses of 4.77 mV and 0.63 mV, respectively, despite retaining a marginally symmetrical shape.

**Figure 8:**
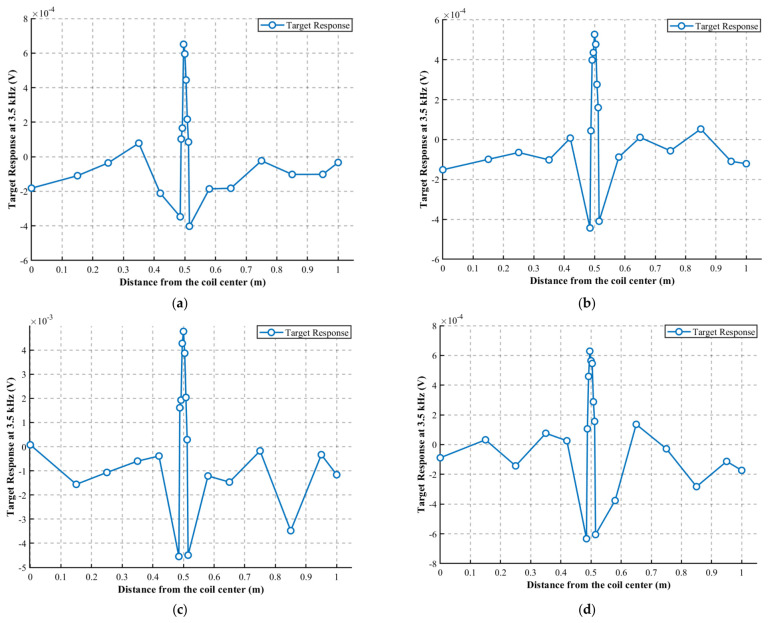

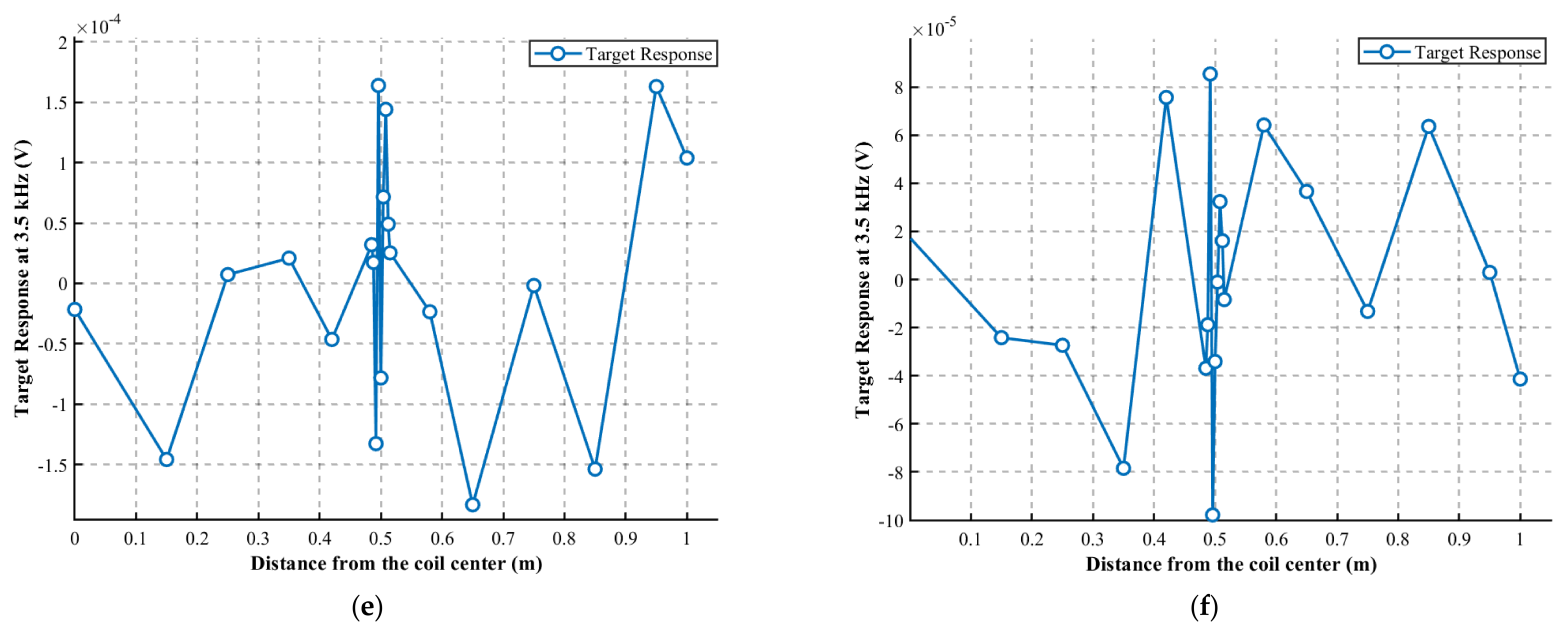
Sensitivity Analysis of Target Physical Properties and Characteristic Frequencies. (**a**) 3.47 kHz. (**b**) 3.53 kHz. (**c**) 1.167 kHz. (**d**) 5.83 kHz. (**e**) 2 kHz. (**f**) 5 kHz.

Extracting at arbitrary non-transmission frequencies such as 2 kHz and 5 kHz resulted in entirely chaotic, featureless noise floors. As shown in Figure 8, these multi-frequency extraction comparisons experimentally confirm that the true Debye relaxation peak of the high-carbon steel wire snare precisely aligns with our algorithmically selected 3.5 kHz, with a deviation margin of less than 10 Hz. Consequently, building the transmission optimization strategy around this characteristic frequency is not only theoretically founded but also highly robust in practice.

## 4. Discussion

This paper proposes a spectrum modification method based on the IR-RIME-SQP algorithm for the transmitting scheme of shallow subsurface electromagnetic detection in forested areas. By coupling the Debye relaxation characteristics of ferromagnetic targets with the VRM colored clutter model of magnetic soils, a multi-objective fitness function guided by SCR maximization was constructed.

### 4.1. Theoretical Universality and Signal Detection Equivalence

The reliability of the underlying physical models is supported by established paradigms in electromagnetic detection. Studies by Cowan et al. and Miller et al. confirm that the Debye model accurately captures the broadband frequency-domain polarization tensor of highly conductive and permeable metal targets, while the VRM clutter exhibits a highly predictable linear evolution with frequency. This implies a natural physical misalignment between the target’s Debye polarization band and the soil’s linear VRM response band.

According to signal detection theory, the fundamental physical prerequisite for improving real-world metrics like detection depth and Receiver Operating Characteristic (ROC) performance in strong VRM environments is enhancing the baseline SCR before the signal reaches the detector. Because the amplitude of soil VRM clutter grows linearly with frequency, injecting arbitrary high-frequency energy will continuously inflate the denominator of the SCR equation. By physically restricting the transmission of non-target high-frequency harmonics, the generation of high-frequency VRM soil clutter is structurally blocked at the source. Thus, verifying the stable emission of the 3.5 kHz bell-shaped spectrum in our experiments is physically equivalent to verifying the suppression of the soil clutter source, establishing a superior baseline for SCR enhancement.

### 4.2. Computational Cost and Convergence Behavior

While the IR-RIME-SQP algorithm introduces a higher computational cost compared to traditional analytical methods, this overhead is exclusively confined to the offline design phase. Standard heuristic algorithms rely on fast analytical Fourier series that ignore transient circuit behaviors. Conversely, IR-RIME-SQP utilizes discrete time-domain ODE integration to simulate true physical current responses, ensuring strict adherence to hardware safety margins. Coupled with the minimax variance regularization strategy that evaluates four physical boundary cases per iteration, the search-phase computational load increases. However, once the optimal robust switching sequence is derived globally, it is compiled into a floating-point lookup table. Consequently, the online execution cost during real-time field detection is identical to that of a simple square wave or PRBS, imposing zero computational latency on the hardware.

Regarding convergence, IR-RIME-SQP overcomes the dead time penalty traps that frequently stall traditional algorithms. The hard-rime puncture strategy allows particles to escape local minima via large-step stochastic jumps, successfully navigating the complex physical constraints. In the final stage, the SQP solver utilizes the Lagrangian–Hessian matrix to execute deterministic steepest-descent cuts, avoiding the random stagnation common to pure meta-heuristics at the tail end of convergence.

### 4.3. Comparison of Waveform Optimization Strategies

To elucidate the specific advantages of the proposed methodology, Table 1 summarizes a comparison between IR-RIME-SQP and previously reported waveform optimization strategies in the literature.

### 4.4. Experimental Limitations

We explicitly acknowledge that the current experimental validation phase faces limitations. Utilizing an RC equivalent circuit based on time-constant duality is a safe workaround for high-current testing, but it fundamentally cannot capture the non-linear magnetic saturation, mutual inductance, and true electromagnetic coupling that a real physical coil experiences with magnetic soil. In addition, the spatial displacement tests were conducted in the air. Following the established research paradigm in shallow surface electromagnetic transmitter design [7,36,37,38], this study deliberately bounds its scope to waveform design and spectral modification at the transmitter end. Therefore, evaluating full-system statistical metrics—such as detection probability, exact false alarm rates, maximum detection depths, and ROC curves—necessitates buried target tests within actual VRM-prone environments integrated with backend adaptive filtering and depth inversion algorithms, which falls outside the current scope.

## 5. Conclusions

This paper proposes a spectrum modification method based on the IR-RIME-SQP algorithm for the transmitting scheme of shallow subsurface electromagnetic detection in forested areas. To implement this scheme, the Debye relaxation characteristics of micro-ferromagnetic targets were coupled with the VRM colored clutter model of magnetic soils to construct a multi-objective fitness function guided by SCR maximization. Furthermore, interval analysis was introduced to effectively overcome the dynamic drift of coil parameters encountered during practical detection. Subsequently, highly robust waveforms were successfully generated subject to the timing constraints of the MCU, and their physical output characteristics were comprehensively evaluated within an RC analog test circuit. According to the simulation and laboratory results, the TFER of the IR-RIME-SQP scheme reaches 51.85%. Compared to the PSO scheme, the proposed approach improves the TFER by approximately 16.4%. This effectively circumvents the excessive consumption of low-frequency fundamental energy and the intense geological clutter induced by high-order harmonic leakage. Consequently, the limited transmission power is more rationally concentrated within the characteristic frequency window required for detection, enhancing both the efficiency and precision of detecting metal snaring traps in shallow forested environments.

Moreover, the proposed spectrum modification method is not strictly confined to the detection of forest snaring traps. By flexibly adjusting the characteristic parameters within the fitness function, this methodology exhibits potential for broad application in other controlled-source electromagnetic detection domains, such as UXO clearance and the surveying of concealed underground pipelines.

In future work, to bridge the gap between laboratory conditions and real forest environments, our primary focus will be on constructing a large-scale magnetic sand tank that mimics true VRM characteristics. Coupled with dedicated backend signal processing hardware and algorithms, we will conduct rigorous buried target tests to systematically quantify the system’s ROC curves, detection probabilities, and field detection depths. We also aim to migrate the algorithm’s computational load onto high-performance processors or edge computing platforms to realize the online solving and dynamic reconstruction of frequency-domain characteristics. These targeted enhancements and rigorous field tests will further explore and maximize the inherent advantages of IR-RIME-SQP in spectrum modification, thereby continuously elevating the efficiency and precision of field detection.

## Figures and Tables

**Table 1:** Comparison of waveform optimization strategies.

Method	Spectral Energy Distribution	Primary Application Scenarios	Main Advantages	Limitations in Shallow Forest Environments
**Bipolar Square**	Highly concentrated at the fundamental frequency.	Deep mineral exploration; general EMI detection.	Simple hardware implementation; low dynamic switching losses.	Severe lack of high-frequency energy; fails to extract critical features of micro-targets; extreme energy waste at low frequencies.
**PRBS**	Broadly dispersed; energy equalization across a wide band.	Multi-frequency deep sounding; broadband feature extraction.	Provides wide-spectrum coverage and abundant geometric information.	Low excitation power at the target’s characteristic frequency; inevitably excites intense, linearly increasing VRM soil clutter, degrading the SCR.
**MOPSO**	Equal energy distributed across the fundamental and specific high-order harmonics.	Deep underground probing; UXO detection in uniform media.	Excellent multi-frequency detection efficiency under white noise; uniform multi-point energy.	High-frequency flat energy injects massive power into clutter-dominated regions; lacks robustness against dynamic coil parameter (L, R) drifts.
**Standard PSO**	Partially concentrated at the target characteristic frequency.	Targeted single-/multi-frequency enhancement.	Enhances specific target frequency amplitude compared to flat broadband methods.	Prone to local optima; poor fundamental wave suppression; generates ultra-narrow pulses violating dead time limits; highly vulnerable to environmental parameter drifts.
**IR-RIME-SQP**	Asymmetric bell-shaped window centered at the optimal SCR frequency.	Shallow subsurface detection of ferromagnetic targets in VRM soils.	Maximizes SCR; actively suppresses fundamental energy waste and high-frequency VRM clutter; strictly adheres to hardware constraints; globally robust against dynamic parameter drifts.	Relies on a priori estimations of operating boundaries and offline solving for microcontroller lookup tables.

## Data Availability

Dataset available on request from the authors.

## Abbreviations

The following abbreviations are used in this manuscript:

ATR	Automatic target recognition
DC	Direct current
DE	Differential Evolution
EM	Electromagnetic
EMF	Electromotive force
EMI	Electromagnetic induction
FDEM	Frequency-domain electromagnetic
FFT	Fast Fourier transform
GA	Genetic Algorithms
GWO	Gray Wolf Optimizer
IR-RIME-SQP	Interval Robust hybrid RIME and Sequential Quadratic Programming
IUCN	International Union for Conservation of Nature
MCU	Microcontroller unit
MOPSO	Multi-Objective Particle Swarm Optimization
NCTLNP	Northeast China Tiger and Leopard National Park
PRBS	Pseudo-random binary sequences
PSO	Particle Swarm Optimization
PWM	Pulse width modulation
RC	Resistor–capacitor
SA	Simulated Annealing
SCR	Signal-to-clutter ratio
SNR	Signal-to-noise ratio
SQP	Sequential Quadratic Programming
TFER	Targeted frequency energy retention
UXO	Unexploded ordnance
VRM	Viscous remanent magnetization
WCS	Wildlife Conservation Society
